# Contemporary contestations over working time: time for health to weigh in

**DOI:** 10.1186/1471-2458-14-1068

**Published:** 2014-10-13

**Authors:** Jane Dixon, Gemma Carey, Lyndall Strazdins, Cathy Banwell, Dan Woodman, John Burgess, Michael Bittman, Danielle Venn, Ginny Sargent

**Affiliations:** National Centre for Epidemiology & Population Health, Australian National University, Canberra, Australia; Department of Sociology, University of Melbourne, Melbourne, Australia; Curtin Business School, Curtin University, Perth, Australia; School of Behavioural, Cognitive and Social Sciences, University of New England, Armidale, Australia

## Abstract

**Background:**

Non-communicable disease (NCD) incidence and prevalence is of central concern to most nations, along with international agencies such as the UN, OECD, IMF and World Bank. As a result, the search has begun for ‘causes of the cause’ behind health risks and behaviours responsible for the major NCDs. As part of this effort, researchers are turning their attention to charting the temporal nature of societal changes that might be associated with the rapid rise in NCDs. From this, the experience of time and its allocation are increasingly understood to be key individual and societal resources for health (7–9).

The interdisciplinary study outlined in this paper will produce a systematic analysis of the behavioural health dimensions, or ‘health time economies’ (quantity and quality of time necessary for the practice of health behaviours), that have accompanied labour market transitions of the last 30 years - the period in which so many NCDs have risen sharply.

**Methods/Design:**

The study takes a mixed-methods approach to capture and explain the relationships between work time and health behaviours. It combines: longitudinal analysis of temporal organisation of work in Australia, with the goal of establishing associations between labour timescapes and health behavioursand health time economies; an in-depth qualitative investigation of employee experiences of the perceived impact of their labour timescapes on ‘health time economies’; and, a stakeholder analysis, will uncover whether, how and why (or why not) stakeholders consider health an important dimension- of work and industrial relations policy, and what efforts are being made to mitigate health impacts of work.

**Discussion:**

The study posits that time is a key mechanism through which particular forms of labour market policies impact health. The labour market flexibility agenda appears to be operating as a time re-distributive device: it has supported the removal of regulations that governed ‘the when’ of working time and removed limits over the amount of working time, thus extending by many hours the notion of the ‘standard’ working week and forcing employees to adapt their shared or social times as well as their time for health.

## Background

The onward march of non-communicable disease (NCD) incidence and prevalence is preoccupying almost every nation, the UN system and major economic development agencies such as the OECD, IMF and World Bank [1]. It has become a focal point for debate in the setting of Sustainable Development Goals as the Millennium Development Goal process draws to a close [2]. The now near universality of this particular health transition is encouraging the search for the ‘causes of the cause’ behind the persistence of a handful of health risks and behaviours – obesity, unhealthy eating, insufficient physical activity, smoking and alcohol abuse – responsible for the major NCDs including Type 2 diabetes, cancers, cardio-vascular disease.

As part of this effort, researchers are turning their attention to charting the temporal nature of societal changes that might be associated with the rapid rise in NCDs [3, 4]. In addition to the temporal dimension to analyses typically deployed by epidemiologists (e.g. age, life stage and length of exposure/birth cohort), the social science notion of ‘historical time’, or “the point in history of the society” in which cohort effects are observed [5] (p 86), is proving to be critical to understanding health transitions. Identifying the events which shape socio-cultural and socio-economic conditions becomes indispensable when seeking to understand the question of ‘why now’ do we see disease emergence? The answers to this question throw light on where to intervene to prevent the future spread of health compromising conditions [6].

The concept of time is not simply central to epidemiology and the social sciences. Notions of ‘the harried worker’, work-life balance and time management have entered the public lexicon. The experience of time and its allocation are themselves becoming acknowledged as a key individual and societal resource and capacity [7–9]. Within public health, time is considered a mediator of basic health behaviours, including healthy eating and physical activity [10], which require careful scheduling and appropriate time allocation [11, 12]. If the practice of these health behaviours change when the time economies of individuals and households change, there will be significant flow on effects for chronic disease [13].

In this interdisciplinary study, we seek to produce a systematic analysis of the behavioural health dimensions, or ‘health time economies’, that may accompany labour market transitions of the last 30 years (the period in which so many NCDs have risen sharply). ‘Health time economies’ refers to the quantity and quality of time necessary for the practice of health behaviours.

### Deregulation and labour moderisation

For more than forty years, research has linked adverse health outcomes to particular working time arrangements – permanent work, no work, precarious work, time limited or short-term work. In its evidence synthesis reports The Employment Conditions Network (EMCONET) of the WHO Commission on the Social Determinants of Health identified the strong links to cardiovascular disease of workplace stress, which was more likely to occur among workers with high workplace demands over long hours and few rest breaks [14]. Long work hours were also associated with occupational injuries and accidents, psychological ill-health, muscoloskeletal disorders and unhealthy behaviors. Increased work intensity (having to do more in less time) raises the risk of anxiety and depression among women workers [14]. However, it is not simply having too much work to do that is a health hazard. Job insecurity and downsizing have also been shown to be linked to poor psycho-social health outcomes. The EMCONET noted that precarious work did not simply negatively impact on the employee’s health but that of family members [14].

While the links between work and health are clear, it is widely recognised that our labour markets are in a state of flux [14–17]. Secure labour force participation, with a high degree of employee control, decent income and social status are all changing. In many OECD countries, since the 1980s, systems of employment regulation have shifted from centralized and collective standards towards workplace and individual bargaining [18]. While labour market deregulation takes a number of concrete forms, at its core is the removal of rules and laws that control the actions of individuals and groups who are ‘party to the production of goods and services’ [19, 20]. Hence, deregulation is seen as the removal of external protections for workers, pursued mainly through changes to award systems [21]. Instead of population wide industrial relations provisions administered by government institutions, a proliferation of specific conditions have been negotiated by market actors and employee representatives. Provisions that regulated working time, such as overtime pay rates for long hours of work, penalty pay rates for unsociable hours of work (evenings, weekends and public holidays) and even holidays have been bargained away. Aided by technology and motived by global competition, the focus of managerial control is increasingly on output not hours. This has loosened the upper limits on work time and uncoupled hours worked from wages, further undermining regulatory influence. This aspect of deregulation also changes other dimensions of work time, including the intensity (having to work faster and compress time to meet deadlines), and unpredictability as labour and work hours is fitted closely to demands, which can fluctuate [22]. The concurrent changes in workforce regulation over the last 30 years have combined with the rapid increases in women’s participation rate in many OECD countries to generate households which are labour market active but time poor.

Like other OECD countries, the Australian context in which this study is embedded has seen a move away from ‘rigid’ standards. The centerpiece of marketing efforts to sell the benefits of a deregulated environment has been to frame less rigid workplace arrangements as offering ‘flexibility’. Broadly, work flexibility refers to the ability of workers to make choices influencing when, where, and for how long they engage in work-related tasks [23]. However, the term is also used to reflect the loosening of hiring and firing terms, as well as the expansion of ordinary hours which do not attract penalty rates [24]. This ‘flexible labour’ is thought to enable globally competitive market places. Internationally, flexible labour has been growing for several decades [17, 18, 25, 26]. Across Canada, Japan and most European countries flexible labour now accounts for as much as 30% of total employment [26] and around 15% in Australia. The trend towards flexible labour is set to continue, with new flexibility provisions in place in industrial relations legislation in many countries, Australia included [26].

Flexibility tends to be described as wholly positive – for employers, employees and the economy [27]. However, the positive rhetoric of workplace flexibility is not backed-up by the research. In a meta review of flexibility studies, Allen [28] found that flextime *and* flexiplace policies were more likely to create work interference with home – suggesting a privileging of work activities over other responsibilities and demands. Flexibility appears to facilitate a shift of more time to work activities [29, 30]. For example, flexibility in the form of greater schedule control can further erode boundaries between work and home: “*employers* now have the flexibility and control to prioritize, scale up and unbind work obligations so that work can impinge on all aspects of employees’ non-work time” [31]. As Shore and Wright argue, flexibility has seen the “re-invention of professionals… as units of resource whose performance and productivity must constantly be audited so that it can be enhanced” [32] (p 559).

Overall, deregulation has led to a more fragmented labour market characterized by unequal wage growth, work intensification and pockets of ‘precarious’ workers [20, 21, 33, 34]. In the Australian context, there has been a marked departure from the design of industrial awards based on the needs of employees to those awards based on the economic performance needs of the industrial sector [35]. This shift to sectoral needs has entailed the introduction of numerous forms of unequal treatment across the workforce: for example, provisions permit the uptake of flexible working time conditions by some workers (parents) and not others, and award entitlements like sick leave and annual leave are awarded to some groups of employees (tenured and some contract, Australian citizens) while not extending the same provisions to others (casual, special work visa categories). Even where good working conditions are present, there appears to be a reluctance to take up the workplace entitlements - sick leave, annual leave, career leave and engaging in industrial disputes [36].

Despite this growing interest in labour market change and population health outcomes, little is known about how contemporary labour market shifts are affecting the health time economies – the amount and quality of time devoted to health related behaviours – of individuals, households, peers and other social groupings. What we do know is that ‘working time’ is a major impediment to time for healthy eating, physical activity and regular, sufficient sleep which has itself been linked to obesity [37, 38]. Ulker finds that non-standard hours are linked to self reports of inferior health through in part negatively impacting on exercise and smoking [39], while long hours and high pressure is known to impact leisure and exercise. Emerging research suggests that time poverty may be more important than income poverty as a barrier to regular physical activity [40, 41]. Time poverty has also been linked to poor eating habits [42], with work time ‘spill over’ associated with lower fruit and vegetable intake [43]. Job stress and long work hours cause individuals to seek out convenience food, which is usually less healthy than food prepared at home [44].

We contend that time is a key mechanism through which particular forms of labour market policies impact health. The labour market flexibility agenda appears to be operating as a time re-distributive device: it has supported the removal of regulations that governed ‘the when’ of working time and removed limits over the amount of working time, thus extending by many hours the notion of the ‘standard’ working week and forcing employees to adapt their shared or social times as well as their time for health. This study seeks to unpack the impact of the dilution of working time regulations and the reduction in the workforce covered by regulations (including access to paid leave and holidays, the extensions to the standard day and week) on the health of the nation.

## Methods/Design

The study aims to capture and explain the relationships between work time and health behaviours, testing the theory that when governments deregulate labour markets they destabilise socio-cultural practices, especially food, physical activity and sleep practices, with potential health and well-being consequences.

The study will also determine whether, how and why (or why not) labour market stakeholders are addressing the health implications of de-regulated labour and flexible work conditions. The research has received approval from the Australian National University Human Ethics Committee.

The research will be carried out in three phases:

1. *Longitudinal analysis of temporal organisation of work (labour timescapes) in Australia, followed by establishing any associations between labour timescapes and the ‘health time economies’* of what are considered health promoting behaviours and practices: meal planning, eating together, slow meals, home based meals as opposed to convenience processed meals, structured physical activity, sufficient moderate to vigorous activity and regular sleep patterns.
2. *Investigation of employee experiences* of the perceived impact of their labour timescapes on ‘health time economies’ – including the degree of employee control they feel they have over working conditions, the presence of flexible working conditions in their workplaces, and the employee’s uptake of those conditions and for what reasons.
3. *Stakeholder analysis -* Here we seek to uncover whether, how and why (or why not) stakeholders consider health an important dimension- of work and industrial relations policy, and what efforts are being made to mitigate health impacts of work. Relevant stakeholders include: industrial relations lobbyists and activists, policymakers, politicians and academic employment regulation experts.

### Longitudinal analysis of temporal organisation of work, mapped against trends in health time economies

While the research on job quality has demonstrated clear links with health outcomes, [45], research on relationships between time spent at work and health are not as definitive or well researched [46]. For example, not all research has shown that non-permanent forms of work are bad for health. Permanent employees tend to have higher stress ratings than non-permanent [47], which have significant flow on effects for health. On the other hand, insecure employment has been found to be negatively associated with self-reported health, depression, anxiety and lower levels of physical activity [47–50]. Long hours on their own, particularly when individuals choose to work them, have not been found to impact health [36]. When long hours are combined with intensification, however, we see more clear cut outcomes for health and wellbeing [36]. In addition, for women in long hour and supervisory positions the use of flexible time provisions is not seen as protecting them from work interference into family life [51].

This opaque picture of the relationships between work, time and health likely stems from out-dated categorisations and understandings of labour-market processes and work structures. Follow-up studies from the UK Whitehall longitudinal studies suggest that the nature of employment contracts (e.g. fulltime, part-time, casual) are a poor proxy for employment insecurity and other dimensions of the work-time relationships, which may impact health [47, 52]. This is also supported by a review of employment contracts by the Australian government [53].

Others have argued that existing research is not nuanced enough in its conceptualisation and categorisation of work-time relationships [47]. Such conclusions, alongside the industrial relations policy shifts reported above, suggest that new categorisations of the temporal organisation of work are needed in order to more accurately guide analysis of their intersections with, and impact on, health.

To begin the process of re-conceptualising the temporal nature of work, we have adopted Barbara Adam’s concept of ‘timescapes’. In her account, lived time has different registers including duration, timing (synchronisation/coordination/rhythm) and sequence (order/succession) which come together in various combinations to produce the timescapes of societies and individual lives. The concept of ‘timescapes’ is useful for distinguishing between the complex array of contemporary employment relationships, in which temporal dimensions of work can vary according to:

1. Duration: average weekly working hours of employment;
2. Tenure: the incidence of short term and temporary jobs;
3. Scheduling: the spread of hours across the day and the week, and in particular, night and weekend work;
4. Rhythms: work tempo and intensification; the predictable interaction between work and non work activities;
5. Synchronization: the ability to coordinate schedules with others so as to engage in activities which involve other people, whether co-workers, peers or household members.

**Figure 1:**
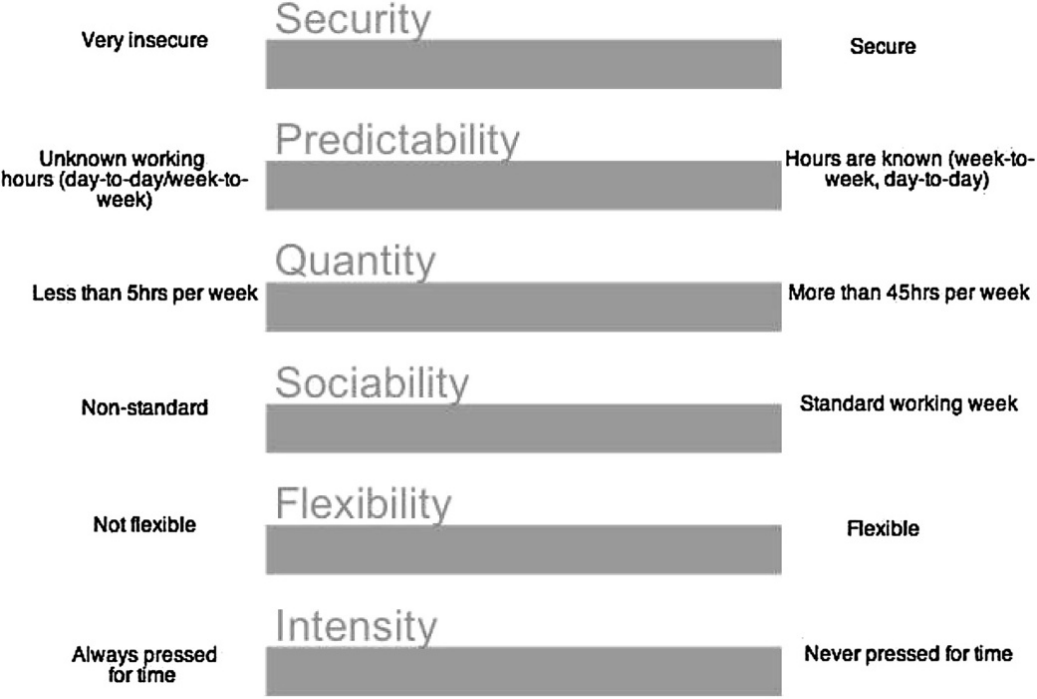
**Working time dimensions instrument.**

Based on this elaboration of ‘labour timescapes’ [9], and an extensive review of labour market literature based on OECD countries, a new Working Time Dimensions instrument has been developed which is more finely orientated to contemporary deregulated labour market’s conditions (see Figure 1). The instrument locates employees along six temporal dimensions of work:

1. Quantity: the number of hours worked
2. Security: how secure employment is.
3. Sociability: how far does employment deviate from the standard working week (e.g. Sundays and/or night work)?
4. Predictability: do employees know in advance what hours they will be working day-to-day or week-to-week?
5. Flexible time: the extent to which employees can vary hours, days, start and finish times
6. Intensity: work tempo and intensification; the predictable interaction between work and non work activities

Data from the Household Income and Labour Dynamics in Australia (HILDA) survey will be used to quantify each of the Working Time Dimensions identified in Figure 1 in order to better understand the true nature of contemporary work. The HILDA survey is funded by the Australian Department of Social Services and administered by the Melbourne Institute of Applied Economic and Social Research at the University of Melbourne. All adult household members in a representative sample of households have been interviewed each year since 2001. The survey covers a broad range of topics, including labour market and family characteristics, income, health and well-being [54].

The analysis will identify which groups are most likely to be affected by poor-quality working time arrangements and how this has changed over time. The Working Time Dimension indices will then be used to examine the links between non-standard working time arrangements and health behaviours, including self-reported physical activity and eating, as well as time pressure, self-rated health and life satisfaction. In particular, we hope to identify which dimensions of working time are most important in influencing health behaviours, and whether these relationships vary by socio-economic status, gender or stage in the lifecourse.

The analysis of historical trends in health time economies will be undertaken alongside the time allocated to labour force participation through using the ABS Time Use Surveys. Time Use Surveys conducted after 1992 contain all members of the household, which will permit the analysis of social coordination of working hours, leisure hours and meal times.

Data on Australian time use is available for 1974 (Cities Commission data on Melbourne and Albury-Wodonga deposited at the Australian Social Science Data Archive at the Australian National University), 1987 (ABS Pilot Survey of Time Use, in Sydney), and 1992, 1997 and 2006 (ABS National Survey of Time Use). Using a method devised to compare the earlier surveys (1974, 1987) with the national surveys allows us to compare broad patterns of time use over a span of 32 years. From 1992 on, ABS time diaries record main activities and secondary at a 3-digit level of detail for all members of the household over the age of 15 years for two consecutive days. The diaries contain information about location and company present when the activity took place. The national surveys also contain information on occupation and industry and weekly work schedule. Diaries give information about work-time patterns and when and in what context eating and activity takes place. Diaries will be subjected to analyses for patterns of engagement in paid work: total hours worked, deviation from Monday-to-Friday 9–5 patterns and variation in hours worked. Eating and physical activity practices will be coded, to cross tabulate with labour market patterns. Having multiple members of the household allows a detailed analysis of the coordination of activities within private dwellings, managing non-parental child care and forms of adult care-giving. Some surveys even collect self-rated health. The SPSS generated analysis of national representative datasets helps interpret the generalizability of conclusions reached through Part 2.

### Investigation of employee experiences

While research on the relationships between work-time structure and healthy practices, such as physical activity and good eating habits, is in its infancy, findings thus far indicate that work schedules, intensity and control impact the health behaviours and outcomes of both individuals and families [43, 44, 55–57]. There is very little experiential work done in this area that examines how individuals and families cope with fractured work-family schedules, time–pressure and intensification in terms of their ability to incorporate healthy eating practices and physical activity.

Employees will be recruited from within two large employers, which have been aproached through Work Cover Victoria, an initiative run by the Victorian State Government aimed at improving the health of employees. The employers represent different economic sectors and hence will have a different of industry awards (health services and construction). The employers have been chosen because they have a diversity of occupations and work structures (e.g. shift work, full-time positions, part-time positions, casual and contract employees) allowing us to cover a range of labour timescapes. Recruitment will be guided by the analysis from Phase 1, and employees will be selected to reflect the categories identified in the new work categorisation typology.

The aim is to provide rich data on the everyday lives of a wide diversity of employees, and to ascertain patterns across particular labour timescapes. Employees will participate in two waves of data collection. Firstly, they will be asked to keep a 48 hour time diary: one 24 hour period is to include what was once considered to be a standard working day and the second 24 hour period is to include Sunday, until 20 or so years ago considered a ‘day of rest’ in Australian society. The diary (adapted from the Longitudinal Study of Australian Children lite time use diary, which itself is adapted from the ABS Time Use Survey) records working and commuting hours, the timing and nature of, and people present at, food-related and physical activity occasions.

The second is an in-depth interview, to be conducted once the diary is completed. Interviews will be organised at the convenience of participants and will be treated confidentially. The diary data will be used as a prompt, along with the Working Time Dimensions instrument (see Figure 1), for a more detailed discussion of labour time and food practices, and to discuss an ‘average’ day in detail. Interview questions will explore the mechanisms by which work patterns shape decisions around the allocation of time to food and physical activity practices, routines and scheduling. Where appropriate the questions asked will mirror the questions used in the HILDA survey (and analysed in Phase 1), so that we can match responses between the qualitative and quantatitive exercises.

Employees do not simply sit on the continua which make up Working Time Dimensions, but they have varying degrees of control over each of these temporal dimensions of work. Four decades of evidence shows that control over working conditions (including time) is predictive of health status [58]. Indeed the control over work pathway – encapsulated by the term ‘flexible work’ - to health outcomes provides a key explanatory foundation for the public health theory of the social gradient in health. To this end, employees will be asked a series of questions regarding flexibility that are adapted from the schema developed by the Centre on Aging and Work at Boston College [23].

Interviews will be audio-recorded, transcribed, coded and sorted according to content, theme and narrative forms using Atlas TI. Coding categories will be determined among the team drawing on previous relevant research while also using an inductive process based on close readings of transcripts. Through investigating employment scheduling, the study will capture experiential data on how work- household interactions affect the routines and synchronization of health time economies, such as shared family meal and structured exercise routines.

Reflecting Adam’s temporal distinctions, practice sociologist Shove [59] poses two overarching questions regarding the temporalities of everyday life which will guide our theoretical interpretation:

- What does the appropriate or competent reproduction of sets of everyday practice, with a focus on healthy eating and physical activity, demand in terms of duration, sequence, coordination and career?
- How do everyday individual temporal profiles emerge, how do they fit together with significant others, and with what [health] consequences?

### Stakeholder analysis

Semi-structured interviews will be undertaken with stakeholders. Criterion-based, purposive sampling of between 10 to 20 individuals chosen on the basis of current/past role in labour market and industrial relations policy, such as industrial relations lobbyists and activists, policymakers, politicians and academic employment regulation experts. In the first instance, the stakeholders will be drawn from WorkCover Victoria’s ‘Work Health Advisory Group’. This group is comprised of a mixture of government, industry and health professionals with an interest in the work-health space. WorkCover will provide the Group with an outline of the project and ask individuals to consent to having their details passed onto the researchers, so they may be contacted about the study.

Questions of stakeholders will be developed once we have undertaken a first pass of analysing the findings form Phases 1 and 2. They will though include issues of how flexibility is conceived, whether it is being used as a rhetorical device, and whether it is mobilised to justify employer and employee practices that bear on healthy lifestyles. The research will also seek to identify stakeholder positions on whether and how health (beyond occupational health and safety) may be viewed as a legitimate principle in industrial relations agendas and the modern award process. Thematic analysis will be conducted on interview transcripts to uncover how health is conceptualised by those in a position to influence work and industrial policy and to identify pathways to elevate health concerns within these debates and amongst stakeholders. Findings will be contrasted with themes identified in the first two phases of the study, with a view to identifying pathways to elevate health concerns within labour market and public health debates and amongst stakeholders.

## Discussion/Conclusion

During the first decade of the 21^st^ century, the World Health Organisation predicted that the obesity epidemic and other chronic conditions would overwhelm health-care budgets and would generate premature mortality amongst younger cohorts around the world. Pessimistically, major reviews of the evidence on obesity prevention and intervention indicate that few behavioural interventions work [1, 60]. As Syme points out: “In intervention study after intervention study, people have been informed about the things they need to do, and they have failed to follow our advice… because they have lives to lead” [6] (pxi).

There is now growing interest in macro-level determinants of health (or the ‘causes of the causes’). Research is this field implores researchers to pay attention to the determinants of health that go beyond medical care and individual interventions [61]. The present study based in Australia, a leader for more than a century in regulating and deregulating its labour market, takes up this challenge; considering how labour market conditions have come – subtly but relentlessly – to foster ways of life that make it increasingly challenging to achieve ‘good health’. The findings from this study will be shared with the European Foundation for the Improvement of Work and Living Conditions as a way of exploring what is common experience across other OECD countries. By examining the operation of national economies as a structural determinant of health through their fashioning of temporal life, the study contributes to a new focus for healthy public policy: linking macro and micro social determinants through an understanding of health practice cultures and time.
